# Dynamics of Sediment Microbial Functional Capacity and Community Interaction Networks in an Urbanized Coastal Estuary

**DOI:** 10.3389/fmicb.2018.02731

**Published:** 2018-11-14

**Authors:** Tianjiao Dai, Yan Zhang, Daliang Ning, Zhiguo Su, Yushi Tang, Bei Huang, Qinglin Mu, Donghui Wen

**Affiliations:** ^1^College of Environmental Sciences and Engineering, Peking University, Beijing, China; ^2^School of Environment and Civil Engineering, Jiangnan University, Wuxi, China; ^3^Institute for Environmental Genomics, Department of Microbiology and Plant Biology, and School of Civil Engineering and Environmental Science, University of Oklahoma, Norman, OK, United States; ^4^Consolidated Core Laboratory, University of Oklahoma, Norman, OK, United States; ^5^State Key Joint Laboratory of Environment Simulation and Pollution Control, School of Environment, Tsinghua University, Beijing, China; ^6^Zhejiang Provincial Zhoushan Marine Ecological Environmental Monitoring Station, Zhoushan, China

**Keywords:** sediment microbial community, metagenomics, functional dynamics, interaction networks, urbanized estuary, inorganic nitrogen

## Abstract

Coastal estuaries and bays are exposed to both natural and anthropogenic environmental changes, inflicting intensive stress on the microbial communities inhabiting these areas. However, it remains unclear how microbial community diversity and their eco-functions are affected by anthropogenic disturbances rather than natural environmental changes. Here, we explored sediment microbial functional genes dynamics and community interaction networks in Hangzhou Bay (HZB), one of the most severely polluted bays on China’s eastern coast. The results indicated key microbial functional gene categories, including N, P, S, and aromatic compound metabolism, and stress response, displayed significant spatial dynamics along environmental gradients. Sensitive feedbacks of key functional gene categories to N and P pollutants demonstrated potential impacts of human-induced seawater pollutants to microbial functional capacity. Seawater ammonia and dissolved inorganic nitrogen (DIN) was identified as primary drivers in selecting adaptive populations and varying community composition. Network analysis revealed distinct modules that were stimulated in inner or outer bay. Importantly, the network keystone species, which played a fundamental role in community interactions, were strongly affected by N-pollutants. Our results provide a systematic understanding of the microbial compositional and functional dynamics in an urbanized coastal estuary, and highlighted the impact of human activities on these communities.

## Introduction

Microbes provide the dominant diversity and biomass in coastal ecosystems, and play a vital role in biogeochemical cycles, climate regulation, and pollutant degradation (Azam and Malfatti, 2007; Lunau et al., 2013; Poi et al., 2013). However, microbial community diversity and functions are highly affected by complex environmental changes. Expanding our knowledge about microbial community dynamics in coastal ecosystems could lead to a better understanding of the response and adaptation of microorganisms to environmental stresses, and further support in-depth evaluation and prediction of human-induced coastal ecosystem variations (Kisand et al., 2012).

As transitional zones of terrestrial, riverine, and marine ecosystems, coastal ecosystems possess strong spatiotemporal heterogeneity in physical and chemical conditions. Numerous studies have suggested that coastal microbial community structures are greatly driven by salinity (Fortunato et al., 2011; Campbell and Kirchman, 2013), depth (Hewson et al., 2007; Boer et al., 2009), nutrients (Wang et al., 2015), dissolved oxygen (DO) (Fortunato et al., 2013; Jeffries et al., 2016), and temperature (Fortunato et al., 2013; Meziti et al., 2016). These factors, which are spatiotemporally dynamic, are crucial to the growth of individual taxa as well as the formation of a given community composition and biogeography pattern. In addition, due to the significant urban and industrial development in coastal areas, they are exposed to intensive input of land-source pollutants (Halpern et al., 2008). Excess pollutants accumulate in coastal water and sediment along with municipal and industrial wastewater discharge, which adds additional stressors to microbial communities (Sun et al., 2012). As a result, polluted areas often present a higher abundance of pollutant-tolerant species and divergent community composition, compared to pristine areas (Sun et al., 2013; Quero et al., 2015; Gubelit et al., 2016). Consequently, identifying microbial dynamics in response to pollutant input at the species or community level is thought to be useful in evaluating possible impacts of anthropogenic disturbance (Aguirre et al., 2017; Richa et al., 2017).

Nevertheless, community composition alone is insufficient in reflecting potential functional variations resulting from environmental changes. Recently, microbial communities and their environmental interactions have been studied both taxonomically and functionally. Network analysis has been proved to be an effective tool in deciphering the species interactions of complex microbial assemblages, since microbial communities generally display non-random and modular network structure that implies strong interactions among functionally related species (Zhou et al., 2011). Thus, the network analysis is thought to be capable of providing information on community system functions rather than the functions of individual species (Ma et al., 2016). Moreover, the microbial community functional dynamics could be characterized by using shotgun metagenomics, which provides direct and valuable functional information presented in the communities’ total DNA. For example, in the highly urbanized Sydney Harbor, microbial functional genes involved in N and P metabolism were closely related to eutrophication due to fertilizer used in agriculture practices, and hydrocarbon degradation genes were possibly stimulated by industrial waste discharge (Jeffries et al., 2016). However, community functional distributions are sometimes less pronounced than taxonomic distributions as the case in Kalamas River (Northwest Greece) (Meziti et al., 2016), and fail to provide effective information about the community dynamics. Therefore, more systematic and accurate understanding of microbial community’s response to environmental changes could be expected by using combined approaches of microbial interaction network and functional genes analysis.

Hangzhou Bay (HZB), located in northern Zhejiang Province, is at the outer reach of the Qiantang Estuary and south of the adjacent Yangtze Estuary. It covers an area of 8,500 km^2^ and a distance of about 100 km from the end of the Qiantang Estuary to the mouth of the bay (Liu et al., 2012). Since the 1980s, the area around HZB has experienced rapid economic growth. There are 14 cities, counties, and districts and 11 large industrial parks around the bay, producing the highest GDP per capita in China. Consequently, the HBZ has been subjected to severe anthropogenic pressures, including land-sourced runoff, municipal and industrial effluents, and maritime engineering. As a result, the coastal environment has significantly deteriorated (Sun et al., 2016). The bay is an ideal area to explore the connections between microbial communities and coastal environments, especially the significant variations in microbial composition and function under long-term anthropogenic stressors.

In this study, we used whole-genome shotgun sequencing and previously reported 16S rRNA gene sequence data to investigate the sediment microbial communities in HZB. Our aims were to (i) characterize dynamic patterns of microbial community functional capacity across environmental gradients in the bay, (ii) identify key environmental drivers and understand how they correlate with community dynamics, and (iii) evaluate the impact of human activities to the coastal microbial community interaction networks. To our knowledge, our results provide the first comprehensive evaluation of sediment microbial compositional and functional dynamics in HZB. As a case study, it also provides important ecological insight into the anthropogenic impacts on microbial communities in an urbanized coastal estuary.

## Materials and Methods

### Sediment Sampling

**FIGURE 1:**
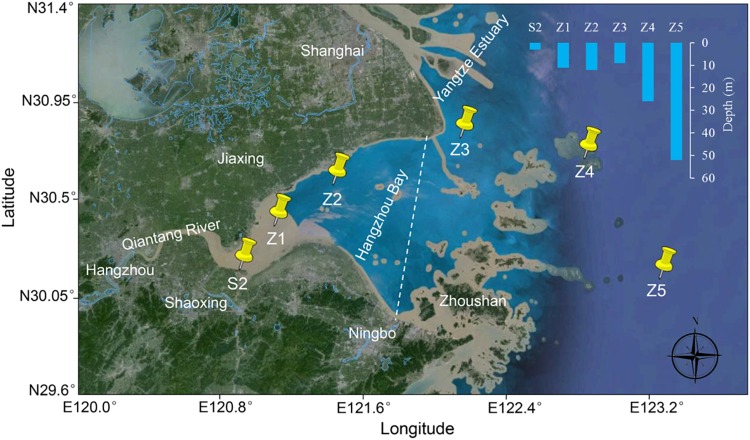
Sampling sites in Hangzhou Bay. A dashed line dividing inner and outer Hangzhou Bay is indicated.

Six sampling sites were designated in Hangzhou Bay from the west inner bay (S2, Z1, and Z2) to the east outer bay (Z3, Z4, and Z5) (Figure 1). For the inner bay sites, S2 is located in the effluent receiving area of a wastewater treatment plant (WWTP) that serves a large fine chemical industrial park (named Shangyu Industrial Area, SYIA) and the urban area of Shangyu County, Shaoxing. Z1 is in the middle of the north and south shore (less than 10 km from both sides), still within the influence of Qiantang River’s runoff and Z2 is close to the Jinshan District, Shanghai on the north shore. For the outer bay sites, Z3 is located at the intersection area of the Yangtze Estuary and Hangzhou Bay. Z4 and Z5 are in the east of Hangzhou Bay, representing a less disturbed sea area.

As previously described (Dai et al., 2016), samples were collected in the spring of 2012 and 2014 at each site. Repeated sampling in the 2 years could improve the reliability in charactering microbial spatial dynamics, since access to more samples from other locations in the bay was limited. Surface sediment (0–5 cm, 1 kg) and its overlying seawater (2 L) were collected in triplicate within a 10 m × 10 m area, and then homogenized thoroughly to be one representative sample at each site. All the samples were stored at -20°C during sampling and transported to the lab within 7 days. The seawater quality was measured immediately in the lab and the sediments were stored at -70°C before DNA extraction.

### Seawater Quality and Sediment Physicochemical Property Analysis

Seawater pH, DO, and salinity were measured in-situ by a pH meter (Thermo Orion 868, United States), a DO meter (Thermo 3-star bench top, United States), and a salinity meter (Mettler Toledo SG3-ELK, Switzerland), respectively. Chemical oxygen demand (COD), ammonia (NH_3_-N), nitrite (NO_2_^-^-N), nitrate (NO_3_^-^-N), and total phosphorus (TP) were analyzed following the standard methods for seawater analysis (GB17378.4-2007) with a UV-VIS spectrophotometer (Shimadzu UV2401, Japan), i.e., alkalescent permanganate titration, indophenol blue, N-1-naphyl-ethylenediamine, alkaline potassium persulfate digestion-zinc cadmium reduction, and potassium persulfate digestion-ammonium molybdate, respectively.

Sediment organic matter (OM) was measured using the K_2_Cr_2_O_7_ oxidation method (Islam and Weil, 1998). Sediment total phosphorus (TP) was measured using Mo-Sb colorimetry (Parker and Fudge, 1927) and total nitrogen (TN) was analyzed using the semi-micro Macro Kjedahl method (Bradstreet, 1954). Sediment pore-water dissolved ammonium (NH_4_^+^), nitrite (NO_2_^-^), nitrate (NO_3_^-^), and sulfate (SO_4_^2-^) were extracted as previously described (Xiong et al., 2014) and measured using a UV-VIS spectrophotometer (Shimadzu UV2401, Japan). For metal content (Cr, Cu, Zn, As, Cd, Pb), air-dried sediment was digested according to standard methods for sediment analysis (GB17378.5-1998), and then measured using an LA-ICP-MS (Agilent ICPMS 7500ce).

### Metagenome Sequencing and Data Processing

DNA samples extracted previously (Dai et al., 2016) were used in this study. One sample from each of the twelve sites was prepared for shotgun sequencing to investigate the microbial functional capacity. However, the library construction for 2012Z5 and 2014S2 failed. For this, total genomic DNA was sheared using the Covaris M220 instrument (Covaris, Woburn, MA, United States). Sequencing libraries (300 bp insert size) were prepared using the TruSeq^TM^ DNA Sample Prep Kit (Illumina Inc.) according to the manufacturer’s instructions and sequenced on an Illumina Hiseq 2000 platform (126 bp paired-end read length).

Raw sequencing data were uploaded to the Metagenomics Rapid Annotation using Subsystems Technology (MG-RAST) server^1^ version 4.0 (Meyer et al., 2008). A quality control process was performed to remove artificial replicate sequences, sequences having Phred score below 15, and sequences with greater than 5 ambiguous bases using dynamic trimming (Cox et al., 2010). After that, protein coding genes were identified using FragGeneScan (Rho et al., 2010), by which coding regions in DNA sequences of 75 bp and longer can be predicted. The identified proteins were clustered at 90% identity level using CD-HIT (Fu et al., 2012) preserving the relative abundances, and the longest sequence for each cluster was used as representative and subjected to similarity analysis against the non-redundant M5nr database (Wilke et al., 2012). Functional annotation was based on the hierarchical classification of SEED Subsystems, with maximum e-value cutoff of 10^-5^, minimum identity of 60% and minimum alignment of 15 bp. The final functional annotation table was normalized to 20,634,952 sequences per sample for downstream statistical analysis. The raw sequencing data are publicly available in MG-RAST (4700815.3, 4700816.3, 4700817.3, 4700818.3, 4700819.3, 4700820.3, 4700821.3, 4700822.3, 4700823.3, and 4700824.3).

### Statistical Analysis

To explore the impact of environmental changes on microbial community dynamics from both compositional and functional aspects, 16S rRNA gene sequencing data of the same samples from our previous study (Dai et al., 2016) was incorporated. Multi-response permutation procedure (MRPP), analysis of similarity (ANOSIM), and non-parametric multivariate analysis (adonis) were used to test the dissimilarity of environmental factors (based on Euclidean distance), community taxonomic structure (based on Sorensen and Bray-Curtis distance), phylogenetic structure (based on unweighted and weighted Unifrac distance), and functional structure (based on Bray-Curtis distance) between sampling years or among locations. Mantel test and Partial Mantel test were used to examine the effect of environmental factors on microbial taxonomic or functional structure dynamics. Pearson correlation was also used to test the relationship between environmental factors and OTUs or functional gene abundances. All analyses were conducted with functions from the Vegan package (2.4-4) in R (v.3.4.3).

### Network Analysis Based on RMT-Based Algorithm

Network analysis is an effective way to understand the interactions between functionally related microbial populations and their responses to environmental changes. The Random Matrix Theory (RMT)-based method (Deng et al., 2012) was used to construct a network for OTUs based on the 16S rRNA gene sequencing dataset. The OTUs detected in less than 8 out of 12 samples were excluded to minimize the impact of rare OTUs. In this study, the cutoff for Pearson correlation coefficient (*R*) between OTUs was determined to be 0.82 based on RMT. Random networks for OTUs were also constructed and compared with the experimental network. The fast-greedy modularity optimization was used to define modules within the network. After network modules were determined, the singular value decomposition (SVD) analysis was used to calculate module eigengene, which described the higher order organizations in the network structure (Deng et al., 2012). The Pearson correlation coefficient between module eigengenes and environmental factors were calculated to explore the response of module members to environmental changes. The network analysis was processed using the pipeline http://ieg4.rccc.ou.edu/MENA/ and visualized in Cytoscape (v.3.6.0) software.

## Results

### Environmental Factors

Environmental factors that describe geographical location, seawater quality and sediment physicochemical properties of the sampling sites are presented in Supplementary Table S1. There were significant (*P* < 0.05) differences of environmental factors spatially, but not between the two sampling years as revealed by three statistical tests, MRPP, ANOSIM, and Adonis (Table 1), indicating the obtained samples should be satisfying in representing the spatial environmental gradients. A mantel test for all environmental factors and the geographical distance indicated significant correlation between these two datasets (*R* = 0.52, *P* = 0.001). Moreover, mantel test detected that salinity (*R* = 0.54, *P* = 0.001), seawater total phosphorus (W_TP, *R* = 0.46, *P* = 0.002) and sediment sulfate (S_Sulfate, *R* = 0.52, *P* = 0.001), were significantly correlated with geographical distance. Particularly, the spatial pattern of seawater quality depicted a typical anthropogenic-interfered coastal environment, i.e., the increase of salinity from site S2 (<4‰) to Z5 (>22‰) indicated the transition from freshwater to saline in the bay. Additionally, the decrease of dissolved ammonia, inorganic nitrogen (DIN), TP, and COD in the seawater implied the input of land-sourced pollutants. Notably, the DIN in the inner bay sites (S2, Z1 and Z2) exceeded the inorganic nitrogen (IN) limit for Class IV of the National Seawater Quality Standard by 3–9-fold (IN ≤ 0.5 mg/L, GB3097-1997). Due to a strong hydrodynamic flow, DO in most sites met the threshold for the Class I standard (DO > 6 mg/L), except S2 and Z5, which may be due to the influence of oxygen consuming compounds and increased water depth, respectively. In addition, the pH was lower in the inner (7.6–8.4) than outer (8.0–8.4) bay sites. The sediment TN and OM were lowest in S2 and Z3, and highest in Z2. Comparably, the content of sediment TP was stable. A significant increase of sediment sulfate was observed in S2 to Z5. Moreover, concentrations of Cr, Cu, Zn, As, Cd, and Pb in the sediment showed strong paired correlations (*p* < 0.05 or even 0.01), and all peak values occurred at Z2, suggesting similar sources of the six heavy metals.

**Table 1:** Dissimilarity test of environmental factors, community taxonomic structure, phylogenic structure, and functional structure.

		Location (inner bay vs. outer bay)						Year (2012 vs. 2014)					
			
		MRPP		ANOSIM		Adonis		MRPP		ANOSIM		Adonis	
									
	Distance measure	δ	*P*	*R*	*P*	*F*	*P*	δ	*P*	*R*	*P*	*F*	*P*
Environmental factors	Euclidean	388.43	0.027^*^	0.36	0.028^*^	8.65	0.014^*^	537.50	0.799	-0.09	0.724	0.31	0.585
Taxonomic structure	Sorensen (unweighted Bray-Curtis)	0.44	0.003^∗∗^	0.39	0.003^∗∗^	3.82	0.003^∗∗^	0.46	0.063	0.17	0.066	1.84	0.083
	Bray-Curtis	0.63	0.043^*^	0.25	0.034^*^	2.24	0.035^*^	0.62	0.028^*^	0.28	0.030^*^	2.34	0.020^*^
Phylogenetic structure	Unweighted Unifrac	0.42	0.009^∗∗^	0.34	0.007^∗∗^	2.84	0.007^∗∗^	0.44	0.087	0.12	0.105	1.56	0.106
	Weighted Unifrac	0.31	0.096	0.14	0.100	2.11	0.051	0.30	0.021^*^	0.21	0.020^*^	2.33	0.030^*^
Functional structure	Bray-Curtis (overall)	0.06	0.119	0.20	0.101	2.11	0.122	0.06	0.136	0.16	0.129	2.22	0.120
	Bray-Curtis (6 categories selected)	0.08	0.035^*^	0.40	0.025^*^	3.52	0.024^*^	0.09	0.316	0.00	0.447	1.12	0.314


*^∗^*P* < 0.05, and ^∗∗^*P* < 0.01.*

### Microbial Functional Dynamics

To explore the sediment microbial functional dynamics, raw unassembled reads from metagenomic sequencing were used for functional annotation. A statistical summary of all the metagenomic libraries is listed in Supplementary Table S2. As shown in Supplementary Figure S1, the gene abundance of each category at level 1 of the SEED hierarchy analysis was consistent among all the samples. Genes encoding core “house-keeping” metabolism pathways predominated in all metagenomes. Gene categories including “Carbohydrates,” “Amino Acid and Derivatives,” “Protein Metabolism,” “Cofactors, Vitamins, Prosthetic Groups, Pigments,” and “RNA metabolism” presented about 44% of all the sequences. A dissimilarity test at the most refined level (level 4) of the whole functional gene profile also revealed no significant variation in the community functional gene composition either spatially or temporally (Table 1).

**FIGURE 2:**
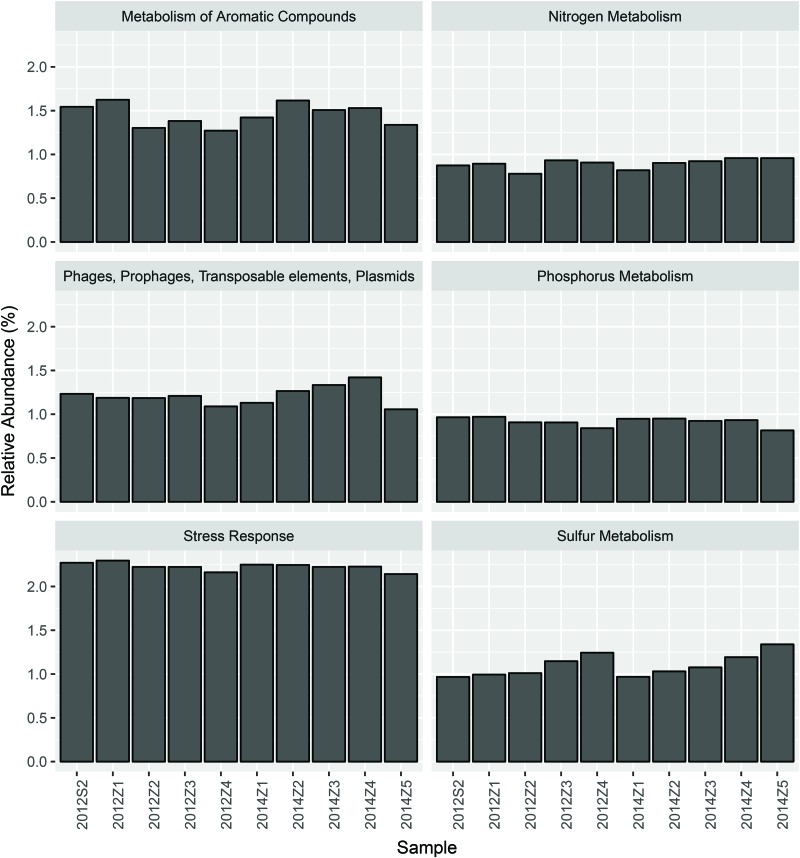
The abundance of 6 selected functional gene categories.

Nevertheless, pairwise comparison identified 7 gene categories exhibiting significant abundance changes between the inner and outer bay, including potassium metabolism, sulfur metabolism, secondary metabolism, regulation and cell signaling, nitrogen metabolism, stress response, and phosphorus metabolism. We selected 4 categories within these (N, S, and P cycling, and stress response), and added another two gene categories, i.e., aromatic compound metabolism, and plasmid and transportable elements, because of their importance in basic biogeochemical cycling and potential in reflecting human-induced environmental stress. As shown in Figure 2, we found the abundance of sulfur metabolism genes increased along the sampling transect from S2 to Z5. The abundance of genes involved in nitrogen metabolism was also higher in the outer than the inner bay sites. In contrast, genes involved in aromatic compound metabolism were in higher abundance in the inner bay sites. A slight decrease in the abundance of phosphorous metabolism genes was also observed from the inner to outer bay. Dissimilarity tests further proved significant differences existed in the selected gene categories between the inner and outer bay, whereas no difference was observed between the years (Table 1).

To coordinate with community functional analysis, we used Sorenson (i.e., unweighted Bray-Curtis) and Bray-Curtis distance to test the community taxonomic dissimilarities, and unweighted and weighted Unifrac distance to test the community phylogenetic dissimilarities, respectively. As shown in Table 1, more significant community compositional variations were observed across the space than between the 2 years. Notably, the community spatial changes were more significant when using unweighted distance measures, indicating the high abundance lineages were more stable than the low abundance ones. However, no significant difference was observed between the years when using unweighted distance measures, indicating the composition of the community phylogenetic lineages between the 2 years was relatively stable.

### The Correlation Between Microbial Communities and Environmental Factors

A Simple Mantel test and a Partial Mantel test indicated that changes in environmental factors, rather than geographic distance, were significantly correlated with the community taxonomic (*R* = 0.45, *P* = 0.002) and phylogenetic (*R* = 0.51, *P* = 0.001) structures (Supplementary Table S3). However, no significant correlation was observed between the community’s overall functional structure and environmental changes or geographic distance (Supplementary Table S3). A Partial Mantel test, by which the effect of spatial distance was controlled, was further used to identify the most influential environmental factors on the microbial community dynamics. As listed in Table 2, seawater pH, salinity, and sediment sulfate content, as well as seawater pollutants including ammonia, DIN, and TP, were significantly correlated with the community taxonomic and phylogenetic structures. Compared with the weighted distance measures, stronger correlations were observed between the unweighted distance measures of community structure and environmental factors. Notably, seawater ammonia (*R* > 0.7, *P* < 0.01) and DIN (*R* > 0.8, *P* < 0.001) were identified as the most closely related environmental factors to the community compositional dynamics. However, the community overall functional dynamics were only significantly correlated with seawater salinity (*R* = 0.39, *P* = 0.02), and the six selected gene categories were correlated with salinity (*R* = 0.47, *P* = 0.004) and COD (*R* = 0.29, *P* = 0.036).

**Table 2:** The relationships between microbial communities and environmental factors revealed by partial Mantel test.

Environmental factors	Community structure						Community function					
		
	Weighted Bray-Curtis		Sorensen (unweighted Bray-Curtis)		Weighted Unifrac		Unweighted Unifrac		Overall		6 categories Selected	
						
	*R*	*P*	*R*	*P*	*R*	*P*	*R*	*P*	*R*	*P*	*R*	*P*
pH	0.41	0.003^∗∗^	0.67	0.001^∗∗∗^	0.34	0.032^∗^	0.65	0.001^∗∗∗^	0.14	0.245	0.10	0.324
DO	0.16	0.138	0.36	0.013^∗^	0.08	0.242	0.41	0.010^∗∗^	-0.20	0.909	-0.21	0.910
Salinity	0.46	0.002^∗∗^	0.40	0.004^∗∗^	0.43	0.004^∗∗^	0.36	0.006^∗∗^	0.39	0.020^∗^	0.47	0.004^∗∗^
COD	0.13	0.153	0.18	0.108	0.10	0.243	0.18	0.119	0.23	0.065⋅	0.29	0.036^∗^
W_Ammonia	0.36	0.033^∗^	0.78	0.003^∗∗^	0.24	0.121	0.76	0.003^∗∗^	-0.21	0.834	-0.24	0.921
W_DIN	0.35	0.024^∗∗^	0.87	0.001^∗∗∗^	0.23	0.179	0.85	0.001^∗∗∗^	-0.15	0.662	-0.16	0.698
W_TP	0.21	0.050^∗^	0.31	0.033^∗^	0.20	0.103	0.25	0.046^∗^	0.09	0.238	0.19	0.114
S_TP	-0.06	0.603	0.33	0.080	-0.03	0.489	0.32	0.094⋅	-0.21	0.932	-0.25	0.974
S_TN	0.15	0.175	0.20	0.165	0.16	0.216	0.17	0.188	0.02	0.424	-0.07	0.635
S_Nitrate	0.09	0.297	-0.05	0.488	-0.02	0.420	-0.04	0.470	0.03	0.369	0.00	0.412
S_Sulfate	0.46	0.001^∗∗∗^	0.49	0.001^∗∗∗^	0.29	0.018^∗^	0.45	0.001^∗∗∗^	0.01	0.445	0.09	0.298
S_OM	0.33	0.026^∗^	0.50	0.005^∗^	0.25	0.077⋅	0.48	0.012^∗^	-0.15	0.766	-0.23	0.935
S_Metal	0.16	0.152	0.21	0.129	0.22	0.119	0.23	0.125	0.04	0.397	-0.07	0.625


*The effects of spatial distance were controlled. Seawater and sediment parameters are prefixed with “W_” and “S_”, respectively (hereinafter inclusive). ^∗^*P* < 0.05, ^∗∗^*P* < 0.01, and ^∗∗∗^*P* < 0.001.*

**FIGURE 3:**
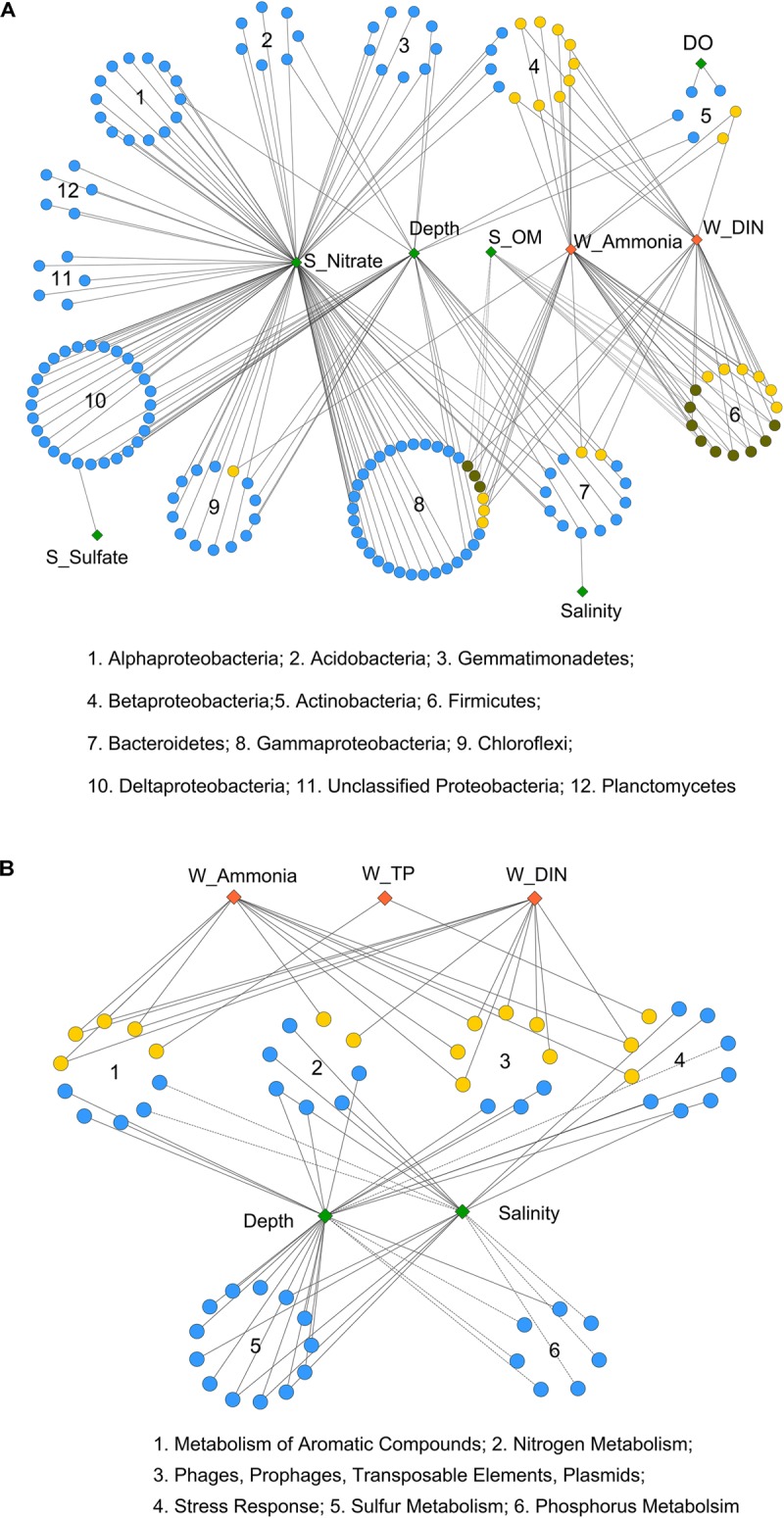
Pearson correlation of environmental factors with **(A)** bacterial OTUs and **(B)** functional genes from 6 selected gene categories. Each diamond represents an environmental factor. The pollutant factors are in red, and the non-pollutant factors are in green. Each circle in **(A)** represents a bacterial OTU, and in **(B)** represents a functional gene (at level 4 of SEED hierarchy). OTUs from the same bacterial phylum (or class for Proteobacteria), and functional genes from the same gene categories (at level 1) are in circular distribution. OTUs or functional genes that only correlated with non-pollutant factors are in blue, only correlated with pollutant factors are in yellow, and that correlated with both are in dark green. Solid and dashed lines indicate significant (*P* < 0.05) positive or negative correlations, respectively. Correlation *p*-values were adjusted using fdr method.

Moreover, Pearson correlation was used to test the correlations between each environmental factor and the abundance of bacterial OTUs or functional genes. As shown in Figure 3A, the bacterial OTUs categorized as Alpha-, Delta-, and Gammaproteobacteria as well as Acidobacteria, Gemmatimonadetes, Chloroflexi, and Planctomycetes were positively correlated with sediment nitrate content. The OTUs classified as Firmicutes, Betaproteobacteria, and some of the Gammaproteobacteria (Pseudomonadales) were positively correlated with seawater ammonia and DIN. While, a few OTUs affiliated with Firmicutes and Betaproteobacteria were negatively correlated with sediment OM content. Additionally, significant correlations were observed between depth and several OTUs classified as Chloroflexi, Delta- and Gammaproteobacteria, Bacteriodetes, Gemmatimonadetes and Acidobacteria. Depth also served as a proxy of other unmeasured physicochemical environmental factors (e.g., light, pressure, and a redox gradient) along the land-sea transect (Caron et al., 2017).

As for overall functional genes categories, seawater salinity and depth were identified as the most influential environmental factors (Supplementary Figure S2). Both positive and negative correlations were detected for gene families involved in basic cellular processes, including “Respiration,” “Protein Metabolism,” and “Clustering-based Subsystems.” The functional genes involved in carbohydrate, nitrogen, and sulfur metabolisms as well as membrane transport were positively correlated with salinity and depth, implying the enhancement of these processes from the inner to the outer bay. In contrast, negative correlations were observed for phosphorus and aromatic compound metabolism genes, implying the enrichment of these genes in the inner bay.

A closer examination of the six selected gene categories is shown in Figure 3B (at level 4) and Supplementary Figure S3 (at level 1). The results further proved that depth and salinity had a substantial impact on these functional gene abundances. Moreover, we found that the seawater pollutants, including ammonia, DIN, and TP, were positively correlated with quite a few genes involved in nitrogen and aromatic compound metabolisms, stress response, and plasmid and transposable elements (Figure 3B). The overall abundances of genes involved in aromatic compound and phosphorus metabolisms also positively correlated with the seawater pollutants (Supplementary Figure S3). These results suggested considerable influences of human induced seawater pollutants on the selected functional gene categories, apart from the influence posed by inherent land-sea environmental gradients.

### Network Interactions of Microbial Community

**FIGURE 4:**
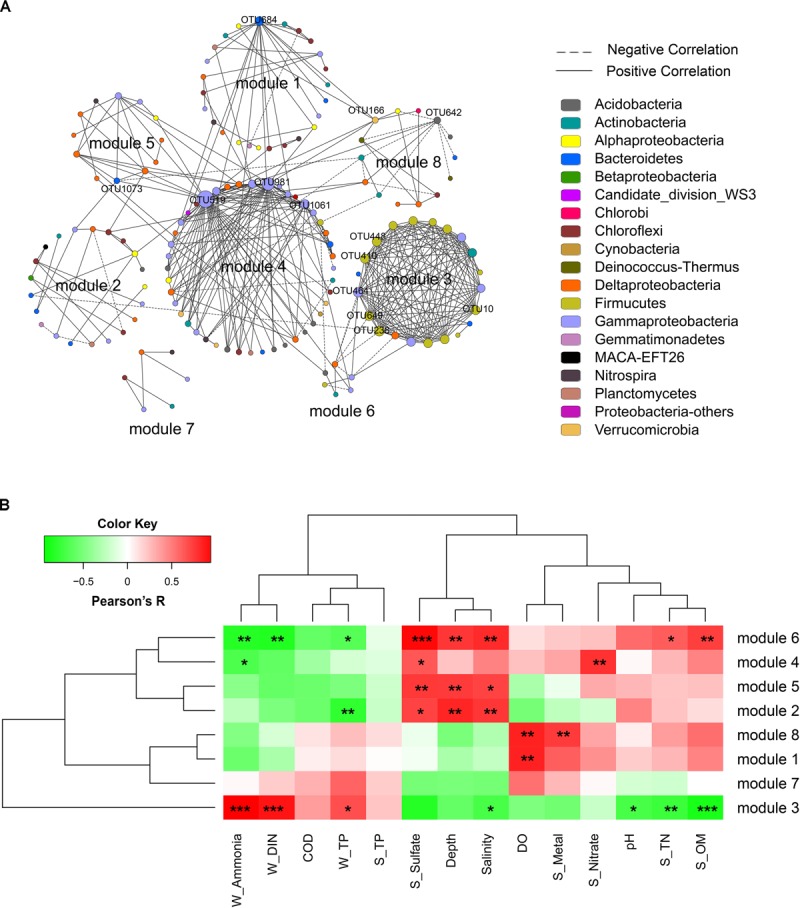
Network analysis of the bacterial community. **(A)** Network of bacterial OTUs at the threshold of 0.82 as determined by random matrix theory (RMT). Nodes represent OTUs, links between nodes indicate significant correlations, and node size indicates degree (i.e., the number of connections). Modules with more than 5 nodes were displayed (8 out of 13 modules). Keystone OTUs (i.e., OTUs with top 10 node degree, or acting as module hubs and connectors) were labeled. **(B)** The correlations between module eigengenes and environmental factors.

A correlation network was generated with a coefficient cutoff of 0.82 as determined by the RMT-based algorithm, in which network nodes represent OTUs and edges represent significant correlations between OTUs (Figure 4A). With higher indices compared to random networks (Supplementary Table S4), the generated network exhibited typical topological features of complex networks, such as scale free, small world, and modularity. Among the 13 modules defined by fast greedy modularity optimization, module 4 contained the most nodes (44 nodes) followed by module 1 (25 nodes). Additionally, module 3 (21 nodes) and module 4 were the most densely connected modules (Figure 4A). Compared to the other modules, the members of these two modules were highly dominated by a few lineages. Of the 21 OTUs (nodes) in module 3, 13 were Bacilli and 4 were Gammaproteobacteria (order Pseudomonadales); and of the 44 OTUs in module 4, 14 were Gammaproteobacteria (mainly from the order Xanthomonadales), 8 were Acidobacteria, and 7 were Deltaproteobacteria (order Desulfobacterales).

The correlations between module eigengenes and environmental factors were used to explore the modules’ response to environmental changes. As shown in Figure 4B, module 3 responded to environmental factors distinctively as compared to other modules, whereas module 2, 5, 4, and 6, and module 7, 1 and 8 were clustered, respectively. Module 3 was positively correlated with ammonia (*R* = 0.94, *P* < 0.001), DIN (*R* = 0.86, *P* < 0.001), and TP (*R* = 0.65, *P* = 0.02); but negatively correlated with seawater pH (*R* = -0.68, *P* = 0.01), salinity (*R* = -0.68, *P* = 0.02), and sediment TN (*R* = -0.72, *P* = 0.009), sulfate (*R* = -0.8, *P* = 0.002), and OM (*R* = -0.85, *P* < 0.001). In contrast, modules 2, 5, 6, and 4 were positively correlated with depth, salinity, and sediment sulfate but negatively correlated with ammonia, DIN, and TP. Additionally, module 1 and module 8 showed a positive correlation with seawater DO. These results indicated that different network modules responded differently to environmental changes, and both the natural and anthropogenic factors had significant impacts on the microbial community interactions.

The topological role of individual network nodes (i.e., OTUs) can be described by the two parameters of *Zi* (within-module connectivity) and *Pi* (among-module connectivity) (Zhou et al., 2011). According to the Z-P plot (Supplementary Figure S4), we identified OTU519, OTU981, OTU684, and OTU642 as module hubs (i.e., OTUs that are highly connected in their own modules), and OTU166 and OTU1073 as connectors (i.e., OTUs that are highly linked to several modules). These topologically important OTUs, together with OTUs with high node degree, played a significant role in the community interactions, and therefore were keystone OTUs of the communities. As shown in Supplementary Table S5, the keystone OTUs in this study were mainly assigned to Gammaproteobacteria, Bacilli, Flavobacteria, Acidobacteria, and Verrucomicrobia. We used the Pearson correlation test to explore the influence of environmental factors on these keystone OTUs. As shown in Supplementary Figure S5, most of the OTUs with top 10 node degree, including OTU448, OTU649, OTU10, OTU238, OTU410 (all affiliated to Bacilli) and OTU464 (Pseudomonadales), were positively correlated with seawater TP, DIN and ammonia, and negatively correlated with pH, salinity, depth, and sediment TN, OM, and sulfate contents. To further determine the extent to which seawater pollutants (W_Ammonia, W_DIN, and W_TP) influenced the keystone OTUs directly, a partial correlation analysis was performed to control for the effect of non-pollutant factors (DO, sulfate, pH, depth, S_OM, S_TN). As shown in Table 3, the influence of ammonia on OTU238, OTU464, OTU448, OTU649, OTU10, and OTU410 remained highly significant (*P* < 0.01) and robust (*R* > 0.75) after controlling any of the non-pollutant factors, and it was the same in most cases with the influence of DIN. These results suggested that seawater inorganic N-pollutants were major factor affecting these OTUs. However, the influence of W_TP were not significant except when DO was controlled (data not shown).

**Table 3:** Partial correlation of network keystone OTUs (i.e., module hubs, connectors, and OTUs with top 10 degree) with seawater ammonia and DIN, in which the effects of non-pollutant factors were controlled.

	OTU519^1^	OTU981^1^	OTU238^3^	OTU464^3^	OTU448^3^	OTU649^3^	OTU1061^3^	OTU684^1^	OTU10^3^	OTU410^3^	OTU642^1^	OTU166^2^	OTU1073^2^
Ammonia | DO	-0.55⋅	-0.31	0.93^∗∗∗^	0.93^∗∗∗^	0.91^∗∗∗^	0.91^∗∗∗^	-0.53⋅	-0.09	0.94^∗∗∗^	0.91^∗∗∗^	-0.66^∗^	-0.45	-0.90^∗∗∗^
Ammonia | Sulfate	-0.49	-0.67^∗^	0.86^∗∗∗^	0.80^∗∗^	0.83^∗∗^	0.84^∗∗^	-0.28	-0.74^∗∗^	0.89^∗∗∗^	0.78^∗∗^	0.24	-0.40	0.40
Ammonia | pH	-0.84^∗∗^	-0.84^∗∗^	0.89^∗∗∗^	0.86^∗∗∗^	0.87^∗∗∗^	0.88^∗∗∗^	-0.77^∗∗^	-0.45	0.91^∗∗∗^	0.86^∗∗∗^	-0.12	-0.53⋅	-0.25
Ammonia | Depth	-0.69^∗^	-0.71^∗^	0.92^∗∗∗^	0.90^∗∗∗^	0.91^∗∗∗^	0.91^∗∗∗^	-0.49	-0.64^∗^	0.94^∗∗∗^	0.88^∗∗∗^	0.00	-0.69^∗^	-0.08
Ammonia | S_OM	-0.58⋅	-0.54⋅	0.84^∗∗^	0.77^∗∗^	0.81^∗∗^	0.81^∗∗^	-0.62^∗^	-0.28	0.88^∗∗∗^	0.75^∗∗^	-0.26	-0.22	-0.34
Ammonia | S_TN	-0.65^∗^	-0.66^∗^	0.94^∗∗∗^	0.87^∗∗∗^	0.90^∗∗∗^	0.89^∗∗∗^	-0.62^∗^	-0.42	0.93^∗∗∗^	0.87^∗∗∗^	-0.24	-0.43	-0.32
Ammonia | Salinity	-0.60⋅	-0.67^∗^	0.89^∗∗∗^	0.84^∗∗^	0.88^∗∗∗^	0.88^∗∗∗^	-0.35	-0.74^∗∗^	0.91^∗∗∗^	0.83^∗∗^	0.09	-0.52	0.11
W_DIN | DO	-0.44	-0.16	0.80^∗∗^	0.81^∗∗^	0.80^∗∗^	0.82^∗∗^	-0.46	0.01	0.87^∗∗∗^	0.74^∗∗^	-0.69^∗^	-0.37	-0.91^∗∗∗^
W_DIN | Sulfate	-0.29	-0.40	0.53⋅	0.46	0.55⋅	0.60⋅	-0.20	-0.55⋅	0.68^∗^	0.37	0.05	-0.22	0.13
W_DIN | pH	-0.81^∗∗^	-0.68^∗^	0.67^∗^	0.74^∗∗^	0.69^∗^	0.73^∗^	-0.87^∗∗∗^	-0.20	0.75^∗∗^	0.66^∗^	-0.33	-0.42	-0.53⋅
W_DIN | Depth	-0.65^∗^	-0.59⋅	0.75^∗∗^	0.74^∗∗^	0.76^∗∗^	0.78^∗∗^	-0.50	-0.54⋅	0.84^∗∗^	0.67^∗^	-0.03	-0.70^∗^	-0.16
W_DIN | S_OM	-0.39	-0.22	0.72^∗^	0.71^∗^	0.73^∗^	0.75^∗∗^	-0.50	-0.03	0.85^∗∗∗^	0.59⋅	-0.46	-0.15	-0.58⋅
W_DIN | S_TN	-0.49	-0.37	0.91^∗∗∗^	0.86^∗∗∗^	0.87^∗∗∗^	0.86^∗∗∗^	-0.51	-0.20	0.93^∗∗∗^	0.80^∗∗^	-0.44	-0.36	-0.56⋅
W_DIN | Salinity	-0.48	-0.48	0.70^∗^	0.63^∗^	0.72^∗^	0.72^∗^	-0.29	-0.65^∗^	0.80^∗∗^	0.55⋅	-0.02	-0.41	-0.04


*Values in the table represent Pearson’s *R*. ^1^Module hub; ^2^Connector; ^3^Top 10 node degree. ⋅*P* < 0.1, ^∗^*P* < 0.05, ^∗∗^*P* < 0.01, and ^∗∗∗^*P* < 0.001.*

## Discussion

The variations of coastal microbial communities along the land-sea transect have been largely described based on taxonomy. In this study, we investigated the microbial community dynamics in HZB using a more comprehensive approach that included both compositional and functional analyses, and explored the key environmental drivers to the community dynamics within this anthropogenically impacted coastal area. We acknowledge that the sample size was relatively small in the present study. Since we observed consistent spatial patterns of environmental parameters during the 2 years, and the community variation was more significant spatially than temporally, the obtained information should be satisfying in capturing microbial spatial dynamics in HZB.

The first goal of this study was to improve our understanding on the microbial community dynamics in the highly urbanized HZB from taxonomic to functional aspect. Our samples covered sharp environmental gradients from inner to outer HZB, and divergent community composition was observed as previously reported (Dai et al., 2016), hence variant functional capacities were expected. Although functional stability was true when considering the overall community functional gene composition, a selected subset of functional genes, including N, S, P cycling, aromatic compound metabolism, stress response, and transportable elements and plasmids, exhibited significant spatial dynamics (Table 1). Previous studies have frequently reported only slight changes in community functions in urbanized water bodies (Bai et al., 2014; Meziti et al., 2016), whereas significant variations have been reported in areas with severe pollution (Ren et al., 2016), or accidental events such as oil spills (Mason et al., 2014; Handley et al., 2017). Considering the HZB area has long been suffering from intensive wastewater discharge and nutrient-rich river input, differentiation of community functional genes, especially for those involved in pollutant metabolism and stress response, could be attributed to strong anthropogenic disturbances.

As shown by our data, each of the selected gene categories covered only 1–2% of the metagenomic sequences (Figure 2), and fluctuations in the abundance of these genes made very little difference to the overall community functional variation. This is because a majority of the DNA sequences was for microbial functional genes involved in basic cellular activities carried out by “house-keeping” genes that present in most organisms, whilst those genes needed for the utilization of special nutrient resources and specific pollutants like PAHs were in low abundance and are likely restricted to certain lineages (Pedros-Alio, 2012; Sauret et al., 2014). In our previous study, we concluded that the large population of low abundance members was the main cause of the microbial community dynamics under natural and anthropogenic environmental gradients of HZB (Dai et al., 2016). In this study, we further confirmed that the low abundance lineages presented higher spatial heterogeneity (Table 1) and stronger correlation with environmental factors (Table 2). Thus, the rare microbial populations, and the small subset of key functional genes are more significant in reflecting the microbial responses to environmental stressors in HZB.

The second goal in this study was to identify key environmental drivers shaping community dynamics. This was explored from both an individual OTU/ functional gene level and from the community composition / functional gene structure level. Seawater DIN, ammonia, and depth, and sediment nitrate were significant in varying the bacterial OTUs (Figure 3A). They also played an important role in determining bacterial community beta-diversity as revealed by a Mantel test (Table 2). However, the community functional variations were only significantly correlated with seawater salinity and depth (Table 2 and Supplementary Figure S2). These two factors are thought to be involved in the early stages of microbial phylogenetic differentiation and dictate whether a microbe can adapt to a given environment (Dupont et al., 2014; Saghai et al., 2016). Thus, the overall microbial functional genes are likely to be more affected by the early evolution of microorganisms, whereas the community taxonomic composition is more sensitive to the current fluctuating environment.

Despite finding no significant correlation for N, P, or S nutrients on overall functional genes, the impact of seawater pollutants (i.e., ammonia, DIN and TP) on the selected gene categories was noticeable, in addition to the influence of depth and salinity (Figure 3B). Strong correlations (*R* > 0.90) suggested a sensitive feedback of these functional genes to divergent environmental stressors. This was possibly underpinned by the selection of adaptive species by pollutants and distinct physical-chemical conditions from the inner to the outer bay. In the inner bay area, Bacilli, Betaproteobacteria, and Pseudomonadales (Gammaproteobacteria) were highly abundant, as they were positively correlated with N-pollutants (Figure 3A). The high abundance of these bacteria in polluted coastal estuaries has been well reported in previous studies (Wu et al., 2010; Peixoto et al., 2011; Meziti et al., 2016). The capability of these species to degrade major organic pollutants in contaminated sites is well recognized (Bassey and Grigson, 2011; Pasumarthi et al., 2013; Koo et al., 2015). Thus, these species were possibly responsible for the enrichment of aromatic compound degradation genes found with increased pollution level. In contrast, the abundance of N and S metabolism genes increased with higher seawater salinity and depth. This was in accordance with a higher abundance of Gammaproteobacteria and Deltaproteobacteria in the outer bay. Xanthomonadales (Gammaproteobacteria, mainly JTB255) and Desulfobacterales (Deltaproteobacteria) dominated the outer bay communities. These bacterial populations prefer high nitrate- and sulfate-habitats (e.g., the outer bay in this study), as they are capable of obtaining energy via N and S metabolism in marine anoxic zones (Matturro et al., 2017; Mussmann et al., 2017). These results support the integrated evidence from community compositional and functional findings, and demonstrate a strong impact of human-induced coastal pollution on key microbial groups and their functional capacities in inner HZB.

Microbial community network analysis provided significant insight into potential interaction of microbial species/populations that are functionally related through flow of energy, matter and information (Zhou et al., 2011), and revealed community responses to environmental changes via module analysis. The influential environmental factors identified above displayed a strong impact on the community interactions. However, the responses of different network modules were distinct. Among the modules, modules 3 and 4 were the most densely connected, indicating stronger metabolic interactions among their members (Luo et al., 2006; Chaffron et al., 2010; Eiler et al., 2012). In addition, the OTUs may have commensal or mutualistic relationships as they were mainly positively correlated (Zhou et al., 2011). Moreover, module 3 was the only module positively correlated with the seawater pollutants (DIN, ammonia, and TP, Figure 3B). This indicated that species in this module, mainly Bacilli and Pseudomonadales, may be deeply involved in the metabolism or catabolism of the pollutants. Specifically, the common genera of *Bacillus* and *Pseudomonas*, who carried genes encoding PAH-catabolic enzymes, have been identified as key “PAH degraders” in soil and sediment (Lu et al., 2011). This may be linked with the enrichment of genes involved in aromatic compounds metabolism in the inner bay. Conversely, species in module 4 might be more active in N and S transformations in the outer bay. This module presented strong positive correlations with sediment nitrate and sulfate contents, and in accordance with the enrichment of functional genes related to N and S metabolisms. Thus, environmental variations substantially influenced the microbial population interactions; such interactions were in close relation to microbial eco-functioning and further propelled the differentiation of microbial community composition along environmental gradients.

Notably, the impact posed by N-pollutants was further supported by their correlation with keystone network OTUs. It has been proposed that keystone taxa are important in maintaining ecosystem functioning, and that their extinction may lead to community fragmentation or even collapse (Wu et al., 2016). The keystone OTUs in this study were mainly categorized as Bacilli and Gammaproteobacteria, which may play important roles in maintaining the coastal microbial functional stability. However, our analysis indicated a strong and robust influence of seawater ammonia and DIN to the keystone OTUs. We noticed that all four module hubs were suppressed by increased N-pollutants, while other keystone OTUs were mostly stimulated. It is possible that high N-pollutant concentrations may have led to a variation in the turnover of microbial interactions and community functioning, as the keystone OTUs were substantially affected by these pollutants. Considering that inorganic nitrogen has long been the primary pollutant in Hangzhou Bay, there is an urgent need for further evaluation of possible ecological risk posed by N-pollutants.

In summary, this study provides both compositional and functional information about the microbial community dynamics along the natural and anthropogenic environmental gradients in HZB. Both community composition and key functional gene categories displayed significant dynamics in response to environmental changes. Besides the strong influence of natural environmental gradients, the impact of human-induced pollution, especially N-pollutants, might play a significant role in determining the coastal microbial diversity and community interaction.

## Author Contributions

TD, YZ, and DW conceived this study. BH and QM performed the sampling. ZS and YT contributed to the physicochemical measurement. TD performed original data analysis and drafted the manuscript. DN contributed to the final manuscript by discussing several essential parts.

## Conflict of Interest Statement

The authors declare that the research was conducted in the absence of any commercial or financial relationships that could be construed as a potential conflict of interest.

## Footnotes

1 http://metagenomics.anl.gov/

## References

[B1] Aguirre M. Abad D. Albaina A. Cralle L. Goni-Urriza M. S. Estonba A. (2017). Unraveling the environmental and anthropogenic drivers of bacterial community changes in the estuary of bilbao and its tributaries. *PLoS One* 12:e0178755. 10.1371/journal.pone.0178755 28594872 PMC5464593

[B2] Azam F. Malfatti F. (2007). Microbial structuring of marine ecosystems. *Nat. Rev. Microbiol.* 5 782–791. 17853906 10.1038/nrmicro1747

[B3] Bai Y. H. Qi W. X. Liang J. S. Qu J. H. (2014). Using high-throughput sequencing to assess the impacts of treated and untreated wastewater discharge on prokaryotic communities in an urban river. *Appl. Microbiol. Biotechnol.* 98 1841–1851. 10.1007/s00253-013-5116-2 23912119

[B4] Bassey D. E. Grigson S. J. W. (2011). Degradation of benzyldimethyl hexadecylammonium chloride by *Bacillus niabensis* and *Thalassospira* sp isolated from marine sediments. *Toxicol. Environ. Chem.* 93 44–56. 10.1080/02772248.2010.504357

[B5] Boer S. I. Hedtkamp S. I. van Beusekom J. E. Fuhrman J. A. Boetius A. Ramette A. (2009). Time- and sediment depth-related variations in bacterial diversity and community structure in subtidal sands. *ISME J.* 3 780–791. 10.1038/ismej.2009.29 19340087

[B6] Bradstreet R. B. (1954). Kjeldahl method for organic nitrogen. *Anal. Chem.* 26 185–187. 10.1021/ac60085a028

[B7] Campbell B. J. Kirchman D. L. (2013). Bacterial diversity, community structure and potential growth rates along an estuarine salinity gradient. *ISME J.* 7:210. 10.1038/ismej.2012.93 22895159 PMC3526181

[B8] Caron D. A. Connell P. E. Schaffner R. A. Schnetzer A. Fuhrman J. A. Countway P. D. (2017). Planktonic food web structure at a coastal time-series site: i. Partitioning of microbial abundances and carbon biomass. *Deep-Sea Res. Part 1-Oceanogr. Res. Pap.* 121 14–29. 10.1016/j.dsr.2016.12.013

[B9] Chaffron S. Rehrauer H. Pernthaler J. von Mering C. (2010). A global network of coexisting microbes from environmental and whole-genome sequence data. *Genome Res.* 20 947–959. 10.1101/gr.104521.109 20458099 PMC2892096

[B10] Cox M. P. Peterson D. A. Biggs P. J. (2010). SolexaQA: at-a-glance quality assessment of illumina second-generation sequencing data. *BMC Bioinformatics* 11:485. 10.1186/1471-2105-11-485 20875133 PMC2956736

[B11] Dai T. J. Zhang Y. Tang Y. S. Bai Y. H. Tao Y. L. Huang B. (2016). Identifying the key taxonomic categories that characterize microbial community diversity using full-scale classification: a case study of microbial communities in the sediments of Hangzhou Bay. *Fems Microbiol. Ecol.* 92:150. 10.1093/femsec/fiw150 27402713

[B12] Deng Y. Jiang Y. H. Yang Y. F. He Z. L. Luo F. Zhou J. Z. (2012). Molecular ecological network analyses. *BMC Bioinformatics* 13:113. 10.1186/1471-2105-13-113 22646978 PMC3428680

[B13] Dupont C. L. Larsson J. Yooseph S. Ininbergs K. Goll J. Asplund-Samuelsson J. (2014). Functional tradeoffs underpin salinity-driven divergence in microbial community composition. *PLoS One* 9:e89549. 10.1371/journal.pone.0089549 24586863 PMC3937345

[B14] Eiler A. Heinrich F. Bertilsson S. (2012). Coherent dynamics and association networks among lake bacterioplankton taxa. *Isme J.* 6 330–342. 10.1038/ismej.2011.113 21881616 PMC3260505

[B15] Fortunato C. S. Eiler A. Herfort L. Needoba J. A. Peterson T. D. Crump B. C. (2013). Determining indicator taxa across spatial and seasonal gradients in the columbia river coastal margin. *Isme J.* 7 1899–1911. 10.1038/ismej.2013.79 23719153 PMC3965310

[B16] Fortunato C. S. Herfort L. Zuber P. Baptista A. M. Crump B. C. (2011). Spatial variability overwhelms seasonal patterns in bacterioplankton communities across a river to ocean gradient. *ISME J.* 6 554–563. 10.1038/ismej.2011.135 22011718 PMC3280145

[B17] Fu L. M. Niu B. F. Zhu Z. W. Wu S. T. Li W. Z. (2012). CD-HIT: accelerated for clustering the next-generation sequencing data. *Bioinformatics* 28 3150–3152. 10.1093/bioinformatics/bts565 23060610 PMC3516142

[B18] Gubelit Y. Polyak Y. Dembska G. Pazikowska-Sapota G. Zegarowski L. Kochura D. (2016). Nutrient and metal pollution of the eastern Gulf of Finland coastline: sediments, macroalgae, microbiota. *Sci. Total Environ.* 550 806–819. 10.1016/j.scitotenv.2016.01.122 26849344

[B19] Halpern B. S. Walbridge S. Selkoe K. A. Kappel C. V. Micheli F. D’Agrosa C. (2008). A global map of human impact on marine ecosystems. *Science* 319 948–952. 10.1126/science.1149345 18276889

[B20] Handley K. M. Piceno Y. M. Hu P. Tom L. M. Mason O. U. Andersen G. L. (2017). Metabolic and spatio-taxonomic response of uncultivated seafloor bacteria following the Deepwater Horizon oil spill. *ISME J.* 11 2569–2583. 10.1038/ismej.2017.110 28777379 PMC5649166

[B21] Hewson I. Jacobson Meyers M. E. Fuhrman J. A. (2007). Diversity and biogeography of bacterial assemblages in surface sediments across the San Pedro Basin, Southern California Borderlands. *Environ. Microbiol.* 9 923–933. 10.1111/j.1462-2920.2006.01214.x 17359264

[B22] Islam K. R. Weil R. R. (1998). A rapid microwave digestion method for colorimetric measurement of soil organic carbon. *Commun. Soil Sci. Plant Anal.* 29 2269–2284. 10.1080/00103629809370110

[B23] Jeffries T. C. Fontes M. L. S. Harrison D. P. Van-Dongen-Vogels V. Eyre B. D. Ralph P. J. (2016). Bacterioplankton dynamics within a large anthropogenically impacted urban estuary. *Front. Microbiol.* 6:1438. 10.3389/fmicb.2015.01438 26858690 PMC4726783

[B24] Kisand V. Valente A. Lahm A. Tanet G. Lettieri T. (2012). Phylogenetic and functional metagenomic profiling for assessing microbial biodiversity in environmental monitoring. *PLoS One* 7:e43630. 10.1371/journal.pone.0043630 22952724 PMC3428334

[B25] Koo H. Mojib N. Huang J. P. Donahoe R. J. Bej A. K. (2015). Bacterial community shift in the coastal Gulf of Mexico salt-marsh sediment microcosm in vitro following exposure to the Mississippi Canyon Block 252 oil (MC252). *3 Biotech* 5 379–392. 10.1007/s13205-014-0233-x 28324540 PMC4522729

[B26] Liu S. Liu Y. Yang G. Qiao S. Li C. Zhu Z. (2012). Distribution of major and trace elements in surface sediments of Hangzhou Bay in China. *Acta Oceanol. Sin.* 31 89–100. 10.1007/s13131-012-0223-y

[B27] Lu X. Y. Zhang T. Fang H. H. P. (2011). Bacteria-mediated PAH degradation in soil and sediment. *Appl. Microbiol. Biotechnol.* 89 1357–1371. 10.1007/s00253-010-3072-7 21210104

[B28] Lunau M. Voss M. Erickson M. Dziallas C. Casciotti K. Ducklow H. (2013). Excess nitrate loads to coastal waters reduces nitrate removal efficiency: mechanism and implications for coastal eutrophication. *Environ. Microbiol.* 15 1492–1504. 10.1111/j.1462-2920.2012.02773.x 22568592

[B29] Luo F. Zhong J. Yang Y. Scheuermann R. H. Zhou J. (2006). Application of random matrix theory to biological networks. *Phys. Lett. A* 357 420–423. 10.1016/j.physleta.2006.04.076

[B30] Ma B. Wang H. Z. Dsouza M. Lou J. He Y. Dai Z. M. (2016). Geographic patterns of co-occurrence network topological features for soil microbiota at continental scale in eastern China. *Isme J.* 10 1891–1901. 10.1038/ismej.2015.261 26771927 PMC5029158

[B31] Mason O. U. Scott N. M. Gonzalez A. Robbins-Pianka A. Baelum J. Kimbrel J. (2014). Metagenomics reveals sediment microbial community response to deepwater horizon oil spill. *Isme J.* 8 1464–1475. 10.1038/ismej.2013.254 24451203 PMC4069396

[B32] Matturro B. Viggi C. C. Aulenta F. Rossetti S. (2017). Cable bacteria and the bioelectrochemical snorkel: the natural and engineered facets playing a role in hydrocarbons degradation in marine sediments. *Front. Microbiol.* 8:952. 10.3389/fmicb.2017.00952 28611751 PMC5447156

[B33] Meyer F. Paarmann D. D’Souza M. Olson R. Glass E. M. Kubal M. (2008). The metagenomics RAST server – a public resource for the automatic phylogenetic and functional analysis of metagenomes. *BMC Bioinformatics* 9:386. 10.1186/1471-2105-9-386 18803844 PMC2563014

[B34] Meziti A. Tsementzi D. Kormas K. A. Karayanni H. Konstantinidis K. T. (2016). Anthropogenic effects on bacterial diversity and function along a river-to-estuary gradient in Northwest Greece revealed by metagenomics. *Environ. Microbiol.* 18 4640–4652. 10.1111/1462-2920.13303 27001690

[B35] Mussmann M. Pjevac P. Kruger K. Dyksma S. (2017). Genomic repertoire of the Woeseiaceae/JTB255, cosmopolitan and abundant core members of microbial communities in marine sediments. *Isme J.* 11 1276–1281. 10.1038/ismej.2016.185 28060363 PMC5437919

[B36] Parker F. W. Fudge J. F. (1927). Soil phosphorus studies I The colorimetric determination of organic and inorganic phosphorus in soil extracts and the soil solution. *Soil Sci.* 24 109–117. 10.1097/00010694-192708000-00004

[B37] Pasumarthi R. Chandrasekaran S. Mutnuri S. (2013). Biodegradation of crude oil by *Pseudomonas aeruginosa* and *Escherichia fergusonii* isolated from the Goan coast. *Mar. Pollut. Bull.* 76 276–282. 10.1016/j.marpolbul.2013.08.026 24045123

[B38] Pedros-Alio C. (2012). The rare bacterial biosphere. *Ann. Rev. Mar. Sci.* 4 449–466. 10.1146/annurev-marine-120710-100948 22457983

[B39] Peixoto R. Chaer G. M. Carmo F. L. Araujo F. V. Paes J. E. Volpon A. (2011). Bacterial communities reflect the spatial variation in pollutant levels in Brazilian mangrove sediment. *Antonie Van Leeuwenhoek* 99 341–354. 10.1007/s10482-010-9499-0 20803251

[B40] Poi E. D. Blason C. Corinaldesi C. Danovaro R. Malisana E. Fonda-Umani S. (2013). Structure and interactions within the pelagic microbial food web (from viruses to microplankton) across environmental gradients in the Mediterranean Sea. *Global Biogeochem. Cycles* 27 1034–1045.

[B41] Quero G. M. Cassin D. Botter M. Perini L. Luna G. M. (2015). Patterns of benthic bacterial diversity in coastal areas contaminated by heavy metals, polycyclic aromatic hydrocarbons (PAHs) and polychlorinated biphenyls (PCBs). *Front. Microbiol.* 6:1053. 10.3389/fmicb.2015.01053 26528247 PMC4602156

[B42] Ren Y. H. Niu J. J. Huang W. K. Peng D. L. Xiao Y. H. Zhang X. (2016). Comparison of microbial taxonomic and functional shift pattern along contamination gradient. *BMC Microbiol.* 16:110. 10.1186/s12866-016-0731-6 27301322 PMC4908767

[B43] Rho M. N. Tang H. X. Ye Y. Z. (2010). Frag gene scan: predicting genes in short and error-prone reads. *Nucleic Acids Res.* 38:e191. 10.1093/nar/gkq747 20805240 PMC2978382

[B44] Richa K. Balestra C. Piredda R. Benes V. Borra M. Passarelli A. (2017). Distribution, community composition, and potential metabolic activity of bacterioplankton in an urbanized mediterranean sea coastal zone. *Appl. Environ. Microbiol.* 83:e00494. 10.1128/AEM.00494-17 28667110 PMC5561294

[B45] Saghai A. Zivanovic Y. Moreira D. Benzerara K. Bertolino P. Ragon M. (2016). Comparative metagenomics unveils functions and genome features of microbialite-associated communities along a depth gradient. *Environ. Microbiol.* 18 4990–5004. 10.1111/1462-2920.13456 27422734 PMC5477898

[B46] Sauret C. Severin T. Vetion G. Guigue C. Goutx M. Pujo-Pay M. (2014). ’Rare biosphere’ bacteria as key phenanthrene degraders in coastal seawaters. *Environ. Pollut.* 194 246–253. 10.1016/j.envpol.2014.07.024 25156140

[B47] Sun M. Y. Dafforn K. A. Brown M. V. Johnston E. L. (2012). Bacterial communities are sensitive indicators of contaminant stress. *Mar. Pollut. Bull.* 64 1029–1038. 10.1016/j.marpolbul.2012.01.035 22385752

[B48] Sun M. Y. Dafforn K. A. Johnston E. L. Brown M. V. (2013). Core sediment bacteria drive community response to anthropogenic contamination over multiple environmental gradients. *Environ. Microbiol.* 15 2517–2531. 10.1111/1462-2920.12133 23647974

[B49] Sun T. Lin W. Chen G. Guo P. Zeng Y. (2016). Wetland ecosystem health assessment through integrating remote sensing and inventory data with an assessment model for the Hangzhou Bay, China. *Sci. Total Environ.* 56 627–640. 10.1016/j.scitotenv.2016.05.028 27236628

[B50] Wang K. Ye X. S. Chen H. P. Zhao Q. F. Hu C. J. He J. Y. (2015). Bacterial biogeography in the coastal waters of northern Zhejiang, East China Sea is highly controlled by spatially structured environmental gradients. *Environ. Microbiol.* 17 3898–3913. 10.1111/1462-2920.12884 25912020

[B51] Wilke A. Harrison T. Wilkening J. Field D. Glass E. M. Kyrpides N. (2012). The M5nr: a novel non-redundant database containing protein sequences and annotations from multiple sources and associated tools. *BMC Bioinformatics* 13:141. 10.1186/1471-2105-13-141 22720753 PMC3410781

[B52] Wu C. H. Sercu B. Werfhorst L. C. V. D. Wong J. Desantis T. Z. Brodie E. L. (2010). Characterization of coastal urban watershed bacterial communities leads to alternative community-based indicators. *PLoS One* 5:e11285. 10.1371/journal.pone.0011285 20585654 PMC2890573

[B53] Wu L. W. Yang Y. F. Chen S. Zhao M. X. Zhu Z. W. Yang S. H. (2016). Long-term successional dynamics of microbial association networks in anaerobic digestion processes. *Water Res.* 104 1–10. 10.1016/j.watres.2016.07.072 27497626

[B54] Xiong J. B. Ye X. S. Wang K. Chen H. P. Hu C. J. Zhu J. L. (2014). Biogeography of the sediment bacterial community responds to a nitrogen pollution gradient in the East China sea. *Appl. Environ. Microbiol.* 80 1919–1925. 10.1128/Aem.03731-13 24413606 PMC3957648

[B55] Zhou J. Deng Y. Luo F. He Z. Yang Y. (2011). Phylogenetic molecular ecological network of soil microbial communities in response to elevated CO2. *Mbio* 2:e122–11. 10.1128/mBio.00122-11 21791581 PMC3143843

