# Supplementary material for: Dynamics of Sediment Microbial Functional Capacity and Community Interaction Networks in an Urbanized Coastal Estuary
Source: Front Microbiol. 2018 Nov 14;9:2731. doi: 10.3389/fmicb.2018.02731 (PMC6246683; doi:10.3389/fmicb.2018.02731)
Supplement: Supplementary file 1 [file Data_Sheet_1.docx]

Supplementary Material

# Dynamics of Sediment Microbial Functional Capacity and Community Interaction Networks in an Urbanized Coastal Estuary

**Tianjiao Dai^1^, Yan Zhang^2^, Daliang Ning^3, 4, 5^, Zhiguo Su^1^, Yushi Tang^1^, Bei Huang^6^, Qinglin Mu^6^, Donghui Wen^1,^ ***

*** Correspondence:**

Dr. Donghui Wen

[dhwen@pku.edu.cn](mailto:dhwen@pku.edu.cn)

**Table S1** Environmental factors at sampling sites

| Samples | 2012S2 | 2012Z1 | 2012Z2 | 2012Z3 | 2012Z4 | 2012Z5 | 2014S2 | 2014Z1 | 2014Z2 | 2014Z3 | 2014Z4 | 2014Z5 |
| --- | --- | --- | --- | --- | --- | --- | --- | --- | --- | --- | --- | --- |
| *Geographic location* |  |  |  |  |  |  |  |  |  |  |  |  |
| Longitude (E) | 120.85 | 121.04 | 121.36 | 122.07 | 122.75 | 123.16 | 120.85 | 121.04 | 121.36 | 122.07 | 122.75 | 123.16 |
| Latitude (N) | 30.21 | 30.43 | 30.62 | 30.85 | 30.75 | 30.17 | 30.21 | 30.43 | 30.62 | 30.85 | 30.75 | 30.17 |
| Depth (m) | 3 | 8 | 9 | 10 | 27 | 60 | 3 | 11 | 12 | 9 | 26 | 52 |
| *Seawater quality* |  |  |  |  |  |  |  |  |  |  |  |  |
| pH | 7.65 | 8.22 | 8.42 | 8.34 | 8.29 | 8.38 | 7.91 | 8.00 | 8.04 | 7.98 | 8.05 | 8.31 |
| DO (mg/L) | 4.35 | 8.10 | 8.39 | 8.20 | 7.03 | 6.02 | 5.28 | 8.17 | 8.26 | 8.12 | 5.78 | 5.43 |
| Salinity (‰) | 1.97 | 3.81 | 4.06 | 13.42 | 24.40 | 20.60 | 3.87 | 9.14 | 11.26 | 13.05 | 18.92 | 22.80 |
| COD (mg/L) | 3.07 | 2.21 | 1.58 | 2.59 | 0.98 | 2.67 | 1.68 | 2.44 | 2.36 | 2.32 | 1.12 | 0.96 |
| Ammonia (mg/L) | 0.32 | 0.07 | 0.06 | 0.04 | 0.01 | 0.03 | 0.35 | 0.04 | 0.00 | 0.03 | 0.01 | 0.02 |
| Nitrate ( mg/L) | 4.14 | 1.34 | 1.41 | 0.68 | 0.39 | 0.18 | 2.72 | 1.39 | 1.35 | 0.79 | 0.37 | 0.09 |
| DIN (mg/L) | 4.47 | 1.42 | 1.47 | 0.72 | 0.4 | 0.21 | 3.07 | 1.43 | 1.35 | 0.82 | 0.38 | 0.12 |
| TP (mg/L) | 0.07 | 0.04 | 0.05 | 0.03 | 0.03 | 0.00 | 0.09 | 0.08 | 0.07 | 0.06 | 0.02 | 0.03 |
| *Sediment physicochemical properties* | | |  |  |  |  |  |  |  |  |  |  |
| TP (%) | 0.05 | 0.06 | 0.06 | 0.05 | 0.06 | 0.05 | 0.07 | 0.05 | 0.06 | 0.05 | 0.05 | 0.05 |
| TN (%) | 0.06 | 0.07 | 0.09 | 0.05 | 0.07 | 0.07 | 0.02 | 0.05 | 0.09 | 0.04 | 0.07 | 0.07 |
| Nitrate (mg/kg) | 19.6 | 15.1 | 18.7 | 19.8 | 9.4 | 15.7 | 14.0 | 26.8 | 37.5 | 55.4 | 31.4 | 29.0 |
| Sulfate (mg/kg) | 59 | 391 | 524 | 495 | 1150 | 1060 | 142 | 420 | 905 | 565 | 1230 | 1260 |
| OM (g/kg) | 4.5 | 11.0 | 14.1 | 8.4 | 13.2 | 10.6 | 4.7 | 10.1 | 14.4 | 7.1 | 11.9 | 11.0 |
| Cr (μg/g) | 46.63 | 72.55 | 89.14 | 82.34 | 72.35 | 53.13 | 57.97 | 72.29 | 77.57 | 66.86 | 79.14 | 58.26 |
| Cu (μg/g) | 14.62 | 17.44 | 39.77 | 35.11 | 21.6 | 13.34 | 12.53 | 31.52 | 30.93 | 18.57 | 26.93 | 12.99 |
| Zn (μg/g) | 45.4 | 55.22 | 103.3 | 84.7 | 70.9 | 59.66 | 44.24 | 78.23 | 85.48 | 61.59 | 85.33 | 65.87 |
| As (μg/g) | 6.17 | 6.98 | 14.37 | 12.61 | 10.3 | 5.96 | 5.32 | 9.78 | 11.97 | 8.97 | 12.17 | 6.11 |
| Cd (μg/g) | 0.12 | 0.18 | 0.24 | 0.20 | 0.16 | 0.11 | 0.17 | 0.20 | 0.20 | 0.14 | 0.16 | 0.10 |
| Pb (μg/g) | 13.99 | 15.73 | 32.24 | 25.67 | 20.80 | 18.89 | 13.42 | 23.01 | 24.73 | 17.96 | 27.83 | 18.57 |

Abbreviations: COD, chemical oxygen demand; DIN, dissolved inorganic nitrogen; TP, total phosphorus; TN, total nitrogen; OM, organic matter.

**Table S2** Overview of the metagenomic sequences and annotations

| Sample | Upload | | |  | Sequence Breakdown | | |  | Predicted Features | | |
| --- | --- | --- | --- | --- | --- | --- | --- | --- | --- | --- | --- |
|  | # of Sequence | Mean Sequence Length (bp) | Mean GC Content (%) |  | Failed QC (%) | Predicted Feature (%) | Unknown (%) |  | Unknown Protein (%) | Annotated Protein (%) | Ribosomal RNA (%) |
| 2012S2 | 88,500,106 | 128 ± 14 | 53 ± 13 |  | 13.27 | 83.57 | 3.17 |  | 66.36 | 24.20 | 9.45 |
| 2012Z1 | 84,861,585 | 129 ± 16 | 55 ± 12 |  | 12.55 | 85.25 | 2.20 |  | 66.01 | 24.95 | 9.04 |
| 2012Z2 | 87,818,029 | 128 ± 15 | 48 ± 13 |  | 16.49 | 75.07 | 8.45 |  | 66.84 | 23.41 | 9.75 |
| 2012Z3 | 90,675,697 | 129 ± 18 | 51 ± 12 |  | 11.75 | 85.40 | 2.84 |  | 67.90 | 23.11 | 8.99 |
| 2012Z4 | 106,501,580 | 129 ± 18 | 49 ± 12 |  | 18.81 | 74.56 | 6.63 |  | 66.53 | 22.81 | 10.66 |
| 2014Z1 | 89,469,355 | 127 ± 12 | 54 ± 13 |  | 13.63 | 82.76 | 3.61 |  | 67.06 | 23.42 | 9.52 |
| 2014Z2 | 94,381,961 | 127 ± 11 | 57 ± 12 |  | 11.78 | 87.31 | 0.91 |  | 67.95 | 23.24 | 8.08 |
| 2014Z3 | 102,303,143 | 130 ± 20 | 54 ± 12 |  | 11.40 | 86.84 | 1.77 |  | 68.10 | 22.94 | 8.96 |
| 2014Z4 | 103,789,327 | 129 ± 17 | 54 ± 12 |  | 10.86 | 88.02 | 1.09 |  | 69.10 | 22.38 | 8.52 |
| 2014Z5 | 92,701,406 | 128 ± 14 | 52 ± 12 |  | 12.26 | 85.09 | 2.65 |  | 68.96 | 22.50 | 8.54 |

**Table S3** The relationship of a microbial community to environmental factors (env) and geographic distance (geo) revealed by Mantel tests and Partial Mantel tests.

|  |  | taxonomic structure | |  | phylogenetic structure | |  | functional structure | |
| --- | --- | --- | --- | --- | --- | --- | --- | --- | --- |
|  |  | *R* | *P* |  | *R* | *P* |  | *R* | *P* |
| Mantel test | env | 0.56 | 0.001*** |  | 0.59 | 0.001*** |  | 0.09 | 0.282 |
|  | geo | 0.41 | 0.024* |  | 0.35 | 0.062 |  | 0.30 | 0.132 |
| partial Mantel test | env (geo) | 0.45 | 0.002** |  | 0.51 | 0.001*** |  | -0.13 | 0.764 |
|  | geo (env) | 0.17 | 0.147 |  | 0.06 | 0.312 |  | 0.31 | 0.127 |

* *P* <0.05, ** *P* <0.01, and *** *P* <0.001

**Table S4** Topological properties of the network for sediment bacterial communities and its associated random networks

| **Experimental Network** |  |
| --- | --- |
| Number of OTUs used for network construction | 246 |
| Network size (n)^a^ | 162 |
| Number of links | 390 |
| Percentage of positive links (%) | 89.0 |
| Modularity (number of modules) | 0.635 (13) |
| *R*^2^ of power-law | 0.94 |
| Average connectivity (avgK) | 4.815 |
| Average clustering coefficient (avgCC) | 0.258 |
| Average path distance (GD)^b^ | 4.574 |
| Harmonic geodesic distance (HD) | 3.498 |
| Centralization of degree (CD) | 0.234 |
| Centralization of betweenness (CB) | 0.458 |
| Centralization of stress centrality (CS) | 2.136 |
| **100 Random Networks** |  |
| Modularity | 0.388 ± 0.008 |
| Average clustering coefficient (avgCC) | 0.101 ± 0.011 |
| Average path distance (GD) | 3.157 ± 0.054 |
| Harmonic geodesic distance (HD) | 2.804 ± 0.034 |
| Centralization of betweenness (CB) | 0.263 ± 0.024 |
| Centralization of stress centrality (CS) | 0.850 ± 0.073 |

^a^The number of OTUs (i.e., nodes) in the network

^b^GD, geodesic distance.

**Table S5** Taxonomic information for keystone OTUs (i.e., module hubs, connectors, and OTUs with top 10 degree).

| OTU ID | Node degree | No. of modules | Phylum | Class | Order | Topological role |
| --- | --- | --- | --- | --- | --- | --- |
| OTU519 | 42 | 4 | Proteobacteria | Gammaproteobacteria | Xanthomonadales | module hub |
| OTU981 | 28 | 4 | Proteobacteria | Gammaproteobacteria | Xanthomonadales | module hub |
| OTU238 | 19 | 3 | Firmicutes | Bacilli | Bacillales |  |
| OTU464 | 18 | 3 | Proteobacteria | Gammaproteobacteria | Pseudomonadales |  |
| OTU448 | 17 | 3 | Firmicutes | Bacilli | Bacillales |  |
| OTU649 | 17 | 3 | Firmicutes | Bacilli | Bacillales |  |
| OTU1061 | 17 | 4 | Proteobacteria | Gammaproteobacteria | Chromatiales |  |
| OTU684 | 16 | 1 | Bacteroidetes | Flavobacteria | Flavobacteriales | module hub |
| OTU10 | 16 | 3 | Firmicutes | Bacilli | Bacillales |  |
| OTU410 | 16 | 3 | Firmicutes | Bacilli | Bacillales |  |
| OTU642 | 9 | 8 | Acidobacteria | Acidobacteria | Subgroup_10 | module hub |
| OTU166 | 7 | 8 | Verrucomicrobia | Verrucomicrobiae | Verrucomicrobiales | connector |
| OTU1073 | 6 | 5 | Bacteroidetes | BD2-2 | norank | connector |

**Figure** **S1** Microbial functional profile at level 1 gene category of SEED hierarchy





**Figure** **S2** Pearson correlation of environmental factors with functional genes. Each diamond represents an environmental factor, each circle represents a level 3 functional gene category of SEED hiearchy. Colors of circles indicate gene categories at level 1. Solid and dashed lines indicate significant (p<0.05) positive or negetive correlations, respectively. Correlation p-values were adjusted using fdr method.


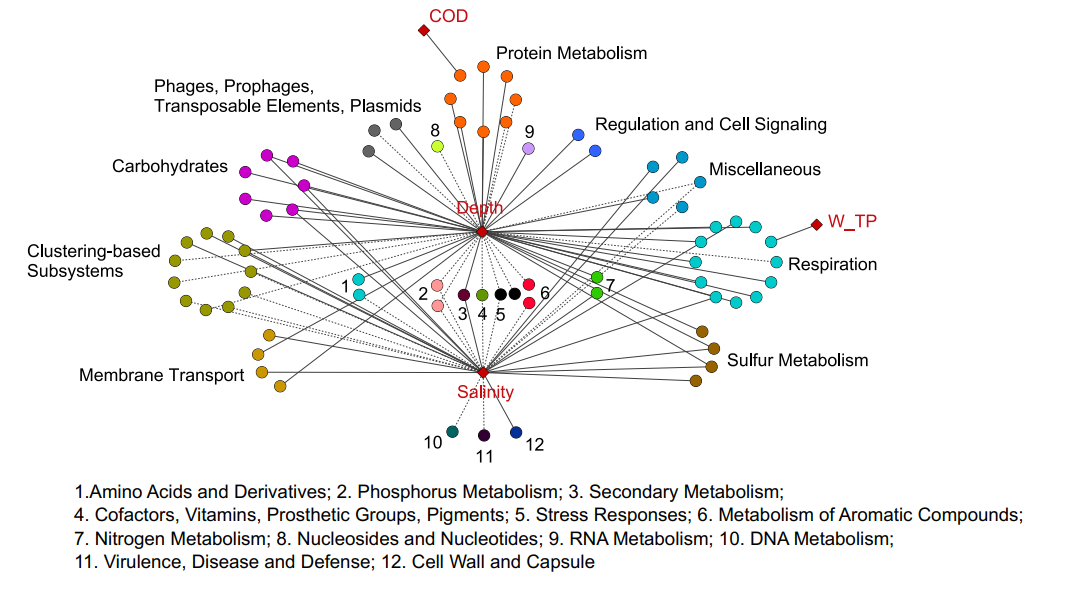


**Figure** **S3** Pearson correlation of environmental factors and the gene abundance at level 1 for the 6 selected gene categories.


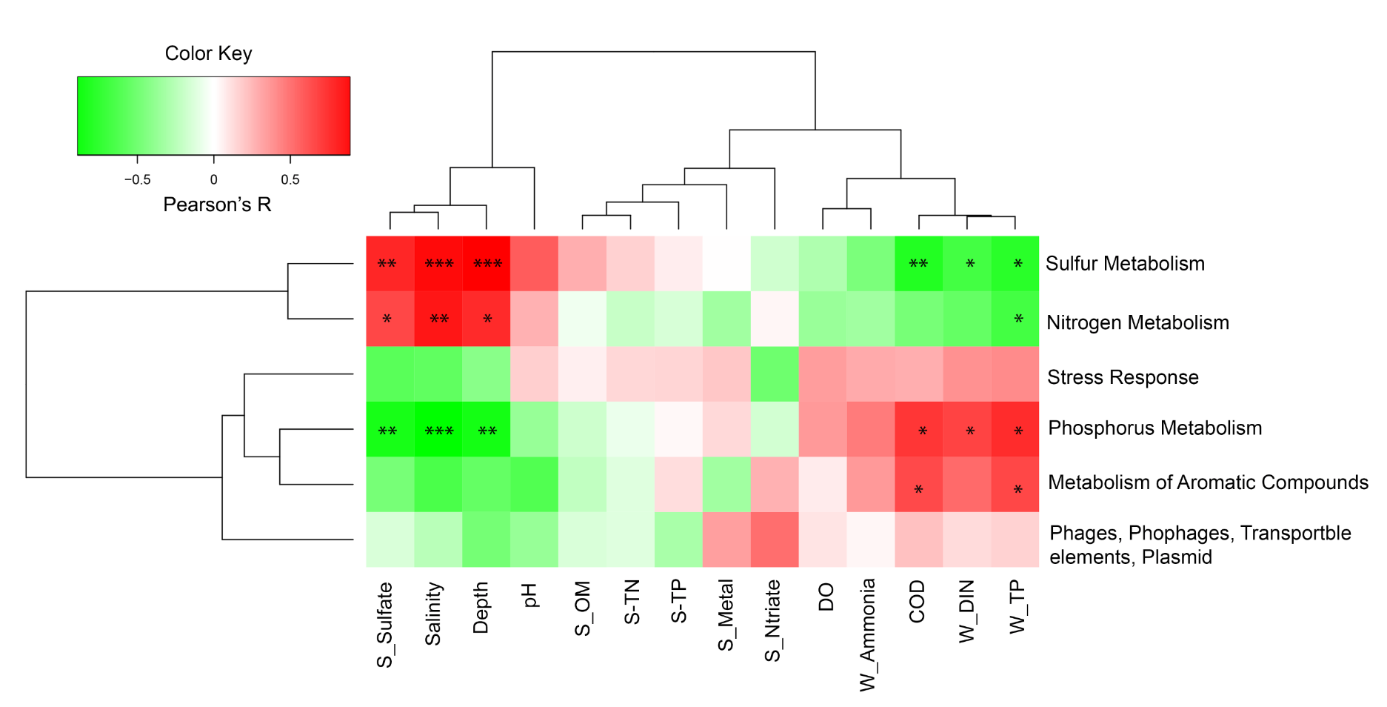


* *P* <0.05, ** *P* <0.01, and *** *P* <0.001

**Figure** **S4** Z-P plot exhibiting the distribution of OTUs based on their topological roles. Pi represents the degree of connections between OTUs from different modules, whereas Zi represent connections between OTUs from same module.


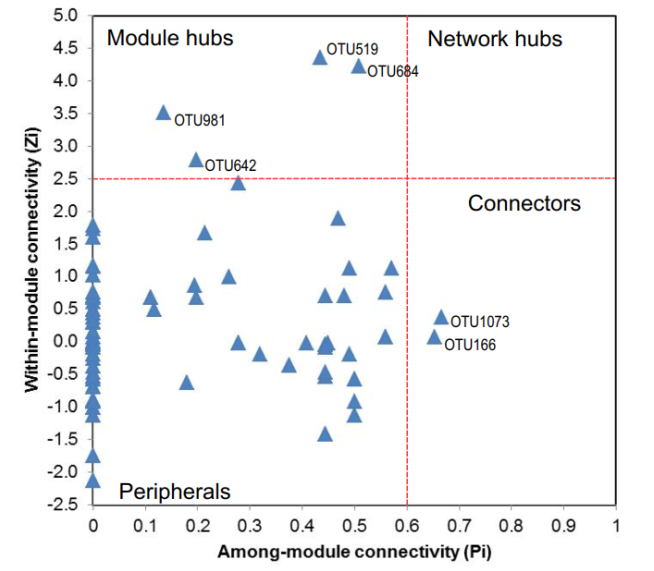


**Figure S5** The relationship between the network keystone OTUs (i.e., module hubs, connectors, and with top 10 node degree) and environmental factors.


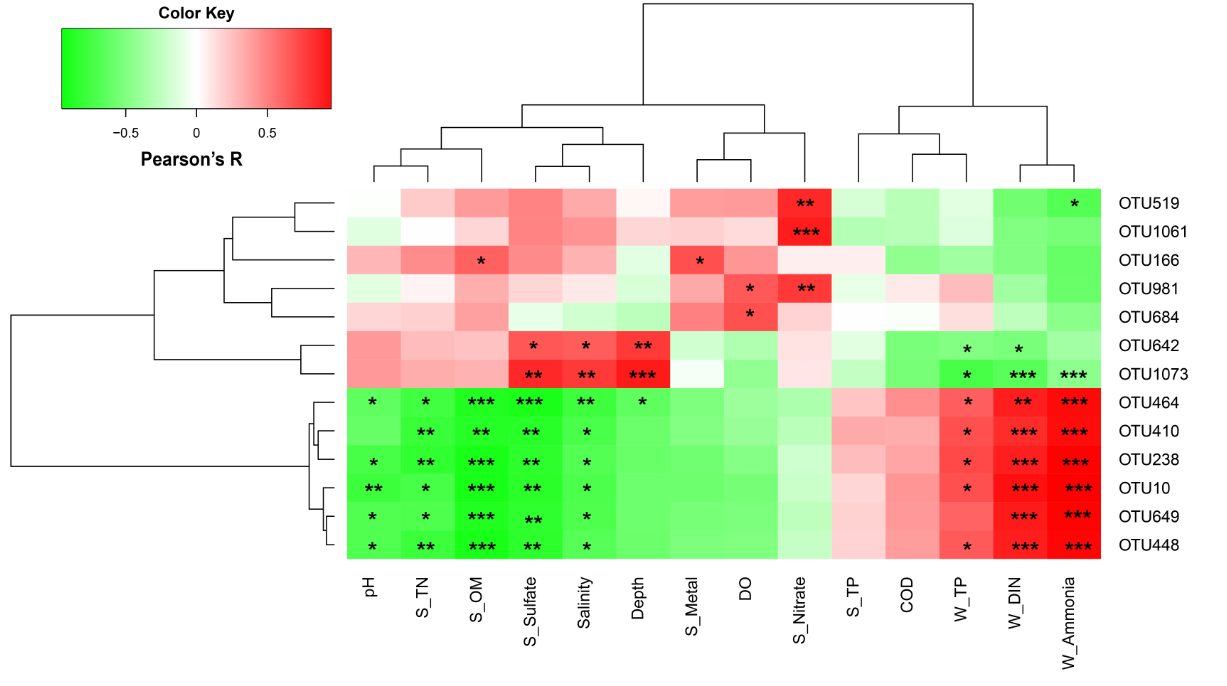


* *P* <0.05, ** *P* <0.01, and *** *P* <0.001
